# Some Characteristic Symptoms

**Published:** 1883-03

**Authors:** 


### SOME CHARACTERISTIC SYMPTOMS.

These symptoms are collected and published, so that those to whom they are not familiar may make note of them. It is a good plan to make immediate record of all good symptoms as soon as found. It is not safe to rely on the memory to retain them.

On lying down, difficult breathing, no constriction of chest; must rise; is afraid to go to sleep; fears suffocation: Baptisia.

Chilly at 11 A. M.; glowing heat in afternoon (in lung troubles): Baptisia.

Wants to be held during chill, shakes so violently: Gels., Lach.

Children peevish, want to be nursed all the time: Ben. ac., Cina, Kreos.

Coffee relieves headache: Cann. ind., Glonoin., Hyos.

Epistaxis, with relief of chest and eyes symptoms: Bromium.

— — —, of backache: Bufo.

After stool great relief, as though an irritating substance had been removed: Gambogia.

Sensation of wave from uterus to throat, which seems to impede labor: Gels.

Bleeding piles; prostration greater than amount of blood would seem to warrant: Hamamelis, Hydras.

Facial neuralgia, with a stupid, stunning headache; begins every morning after breakfast; copious urination and disposition for stool: Iris v.

Skin itches; he scratches until he vomits: Ipec.

Watery, gushing diarrhœa in morning; awakes with violent tenesmus, which prevents her rising; later, burning in abdomen, nausea, and violent straining to vomit: Kali b.

Cold sensation about heart: Kali b.

Pains occur at irregular times, continue for no definite period; come suddenly or gradually and leave as uncertainly: Kalmia.

Pains are worse when sitting bent, yet feels as though it were necessary to do so; relieved by sitting or standing upright: Kalmia.

Pains in back and legs after eating: Kali c.

Sleepy while eating: Kali c.

Has an intoxicated feeling during and after meals: Gratiola.

Constant dripping of blood from the anus (stools not bloody): Kobaltum.

Increased secretion of urine in morning after drinking coffee: Kobaltum.

A tearing sensation in throat, not when swallowing, but from exertion of mind: Caust.

Mucus in throat; fears he will suffocate if he closes his eyes; lessens when opening eyes and sitting up; keeps awake all night: Carbo an.

Least solid food gags; can swallow drinks only: Baptisia.

Only fluids can be swallowed; solids reach a certain point and are then violently ejected: Natr. m.

Copious urine, with headache: Cinnab., Glon., Iris v., Verat.

Profuse micturition relieves headache: Gels., Kalm., Silicea.

— — — backache: Lyc.

— — with dropsical swelling of ankles and feet: Eupat. per.

Diarrhœa (*i. e.*, an increased stool) relieves headache: Agar., Alum., Apis, Lachnan.

Washing chest with cold water relieves chest symptoms: Borax.

Wetting hæmorrhoids with saliva relieves pain, while cold or warm water aggravates: Bromium.

Sensation as if drops of water were falling from the heart: Cann. ind.*

Gnawing pressure in stomach; relieved after eating, but returns as soon as stomach is empty: Lachesis.

Nausea after cold, not after warm, drinks (in "chills"): Lycop.

Fluids swallowed with greater difficulty (and more pain) than solids: Brom., Cocc., Hyos., *Lach.*

Head and uterine pains, ameliorated by flow of blood: Lach.

Sciatica, right side, cannot walk or sit, must lie down: Lach.

All symptoms relieved while walking, after sitting down obscuration of sight returns: Lachnan.

Drinking coffee causes pain in all the teeth: Lachnan.

Diarrhœa, stools of green liquid mucus, with suffocative spells about heart, forcing one to lie down: Lauro.

Black tarry stool, followed by distress in liver; worse morning as soon as moves (Nat. sul.): Leptan.

Pain under right scapula, causing nausea or vomiting: *Chelid.*

When he sits the urine dribbles from him; when he stands it passes freely: Sarsap.

Most horrible erections at night, causing the patient to *swear* most vehemently: Picric. ac.

So much pain when he passes urine as to cause him to *dance* around the room in agony: Petrosel.†

---

* Recently verified clinically.—EDITOR.

† The last three symptoms were given by Dr. C. Carleton Smith, who has clinically verified them.

